# Successful Treatment with Bosentan of Lower Extremity Ulcers in a Scleroderma Patient

**DOI:** 10.1155/2013/690591

**Published:** 2013-04-03

**Authors:** Alix Naert, Petra De Haes

**Affiliations:** Department of Dermatology, University Hospital, KU Leuven, Kapucijnenvoer 33, 3000 Leuven, Belgium

## Abstract

Digital ulcers are a well-known problem in patients with systemic sclerosis. Lower extremity ulcers are less prevalent but are also a challenging and underestimated complication of the disease causing important pain and morbidity. Bosentan, an oral dual endothelin receptor antagonist, has been shown to be effective in preventing digital ulcers in patients with systemic sclerosis. A few recent observations showed the efficacy of bosentan for accelerating the healing of nondigital ulcers in scleroderma patients. This report deals with a 48-year-old patient with systemic sclerosis who developed painful ulcers on the left ankle and hallux. The ulcers were refractory to a combination of vasodilator therapy with a calcium antagonist and several courses of intravenous prostanoids, low molecular weight heparin, aspirin, simvastatin, and intensive local treatment. Bosentan treatment showed spectacular healing of the ulcers already after 4 months of therapy. This case supports the previous few observations of accelerating wound healing of lower extremity ulcers in systemic sclerosis patients with bosentan treatment.

## 1. Introduction

Digital ulcers are a well-known problem in patients with systemic sclerosis with a prevalence around 30%. Lower extremity ulcers are less prevalent but are also a challenging and underestimated complication of the disease causing important pain and morbidity. In a recent clinical study the prevalence of nondigital ulcers in scleroderma patients was estimated at 4% [1].

## 2. Case Report

**Figure 1:**
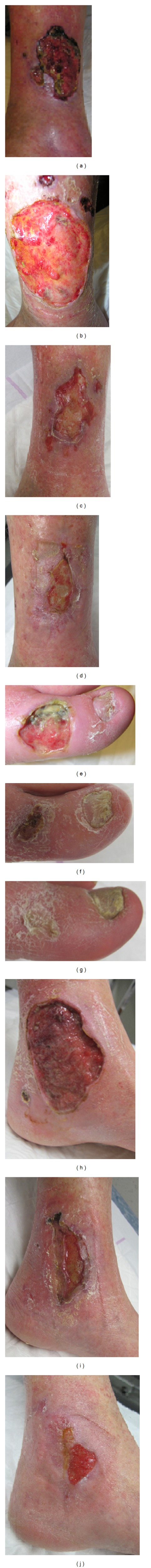
Clinical course of skin ulcers. (a) Skin ulcer with necrotic tissue and cyanotic skin of the left outer ankle 6 months before starting bosentan, (b) at the start of bosentan, and (c) 4 months and (d) 6 months after the administration of bosentan. (e) Skin ulcer of the left hallux 6 months before starting bosentan, (f) after intravenous administration of prostanoids, and at the start of bosentan therapy. (g) Complete healing after 6 months of bosentan therapy. (h) Skin ulcer of the left inner ankle at the start of bosentan, (i) after 4 months (j) and after 6 months of bosentan therapy.

This report deals with a 48-year-old man with a 29-year history of systemic sclerosis, who had a 6-month history of painful ulcers on the inner and outer left ankle and on the left hallux. He already had a history of leg and digital ulcers 4 years earlier who responded quite well to intravenous administration of prostanoids. Besides recurrent ulcers he suffered from sclerodactyly, severe Raynaud phenomenon for which he underwent a sympathectomy in the past, and severe esophageal dysmotility with secondary anorexia. There was no evidence for pulmonal or cardiac involvement. Laboratory evaluation showed antinuclear antibody titer of 1 : 1280 with U1-RNP >240 U/mL and RNP-70 of 104 U/mL. Anti-cardiolipin antibodies were negative. Ankle-branchial index showed no signs of arterial insufficiency. In January 2011 the patient developed a painful, sharply margined necrotic ulcer with purple edges on the left outer ankle (Figure 1(a)) and an ulcer on the left hallux (Figure 1(e)). Intensive local treatments with amniotic membrane transplantation, keratinocyte grafts, and silver foam dressings were unsatisfactory. Use of a dihydropyridine-type calcium antagonist, aspirin, and simvastatin [2] was unsuccessful. In April 2011 he developed a new ulcer on the left inner ankle (Figure 1(h)). Bacterial superinfection occurred and many antibiotic treatments were necessary. Because of further deterioration intravenous administration of prostanoids in combination with subcutaneous administration of low molecular weight heparin [1] was started in May 2011. Only the ulcus on the left hallux responded partially (Figure 1(f)). The leg ulcers however did not show any improvement (Figure 1(b)). Bosentan 62.5 mg twice a day was started and after 4 weeks elevated to a dose of 125 mg twice a day which was tolerated well. Local treatment was continued with silver foam dressings. After 4 months the ulcers got shallow and smaller and showed good granulating tissue and reepithelialization at the wound edges (Figures 1(c) and 1(i)). After 6 months the leg wounds showed further healing (Figures 1(d) and 1(j)), the ulcer on the hallux closed completely (Figure 1(g)), and the patient was free of pain.

## 3. Discussion

The origin of the ulcers in systemic sclerosis is thought to be multifactorial, including microangiopathy, macrovasculopathy, microtrauma, bacterial infection, fibrosis, and calcinosis. Chronic microangiopathy seems to play an important role in the pathogenesis with endothelial cell damage being most probably the initiating factor [3]. Endothelin-1, a highly potent vasoconstrictor produced by the endothelial cells, is believed to be a key mediator of the vasculopathy [3]. Two randomized, double-blind, placebo-controlled, multicentre trials showed that bosentan (Tracleer), an orally administered dual endothelin-1 receptor antagonist, prevents the development of new digital ulcers in patients with systemic sclerosis [4, 5]. Those studies showed no effect on digital ulcer healing. The effect of bosentan on lower extremity ulcers has not been well studied. A recent clinical study [6] (*n* = 5), however, showed the efficacy of bosentan in accelerating the healing of nondigital ulcers surrounded with severe cyanosis. This would suggest that nondigital ulcers caused by severely impaired peripheral circulation are highly responsive to this treatment. Nondigital ulcers without cyanosis, however, were still refractory to bosentan therapy. In the literature we found 2 case reports describing the healing of a lower extremity ulcer in a patient with longstanding systemic sclerosis after 6 months of bosentan therapy [7, 8]. In our patient bosentan treatment showed spectacular healing of the ulcers already after 4 months of therapy. The ulcer on the outer left ankle showed signs of cyanosis 6 months before starting the bosentan treatment. In addition, no new ulcers developed since starting the bosentan treatment. This case supports the previous few observations of accelerating wound healing of lower extremity ulcers in systemic sclerosis patients with bosentan treatment. 

## 4. Conclusion

Lower extremity ulcers in patients with systemic sclerosis, refractory to conventional treatments, can be responsive to treatment with bosentan, especially when the ulcers are surrounded with cyanosis.
